# Chemical systems, chemical contiguity and the emergence of life

**DOI:** 10.3762/bjoc.13.155

**Published:** 2017-08-07

**Authors:** Terrence P Kee, Pierre-Alain Monnard

**Affiliations:** 1 School of Chemistry, University of Leeds, Leeds LS2 9JT, UK,; 2 Institute of Physics, Chemistry and Pharmacy, University of Southern Denmark, Campusvej 55, 5230 Odense M, Denmark

**Keywords:** chemical contiguity, chemical systems, geochemical environment, prebiotic synthesis, protocell

## Abstract

Charting the emergence of living cells from inanimate matter remains an intensely challenging scientific problem. The complexity of the biochemical machinery of cells with its exquisite intricacies hints at cells being the product of a long evolutionary process. Research on the emergence of life has long been focusing on specific, well-defined problems related to one aspect of cellular make-up, such as the formation of membranes or the build-up of information/catalytic apparatus. This approach is being gradually replaced by a more “systemic” approach that privileges processes inherent to complex chemical systems over specific isolated functional apparatuses. We will summarize the recent advances in system chemistry and show that chemical systems in the geochemical context imply a form of chemical contiguity in the syntheses of the various molecules that precede modern biomolecules.

## Review

### Introduction

**Figure 1:**
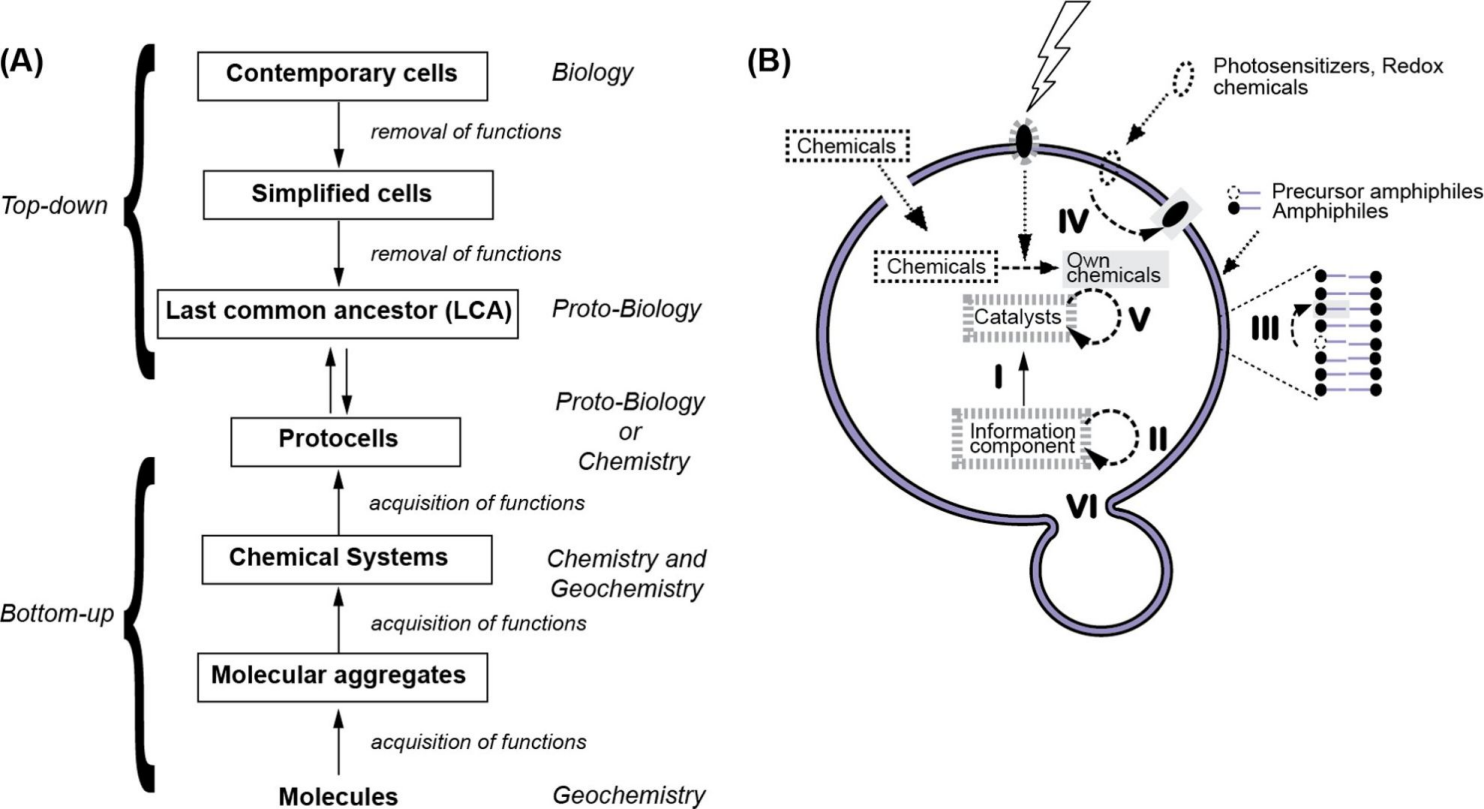
(A) Possible approaches to the historical reconstruction. Two complementary approaches exist: top-down and bottom-up. In the former, the idea is to simplify the cellular architecture and cellular biochemistry by removing redundant or dispensable functions. These are functions that can be either replaced by providing chemicals or taken over by simpler chemicals easily synthesized by, e.g., “non-coded” protein catalysts, or performed, perhaps less efficiently, by other catalysts in the cells. The process should be repeated until a very simple putative “protocell” stage (vide infra) is attained. This is likely a point in time at which biology did not yet exist, but instead pure chemistry defined the protocellular reaction network. The latter approach is based on the use of molecule sets that can self-assemble into chemical aggregates and systems that will then be able to perform an increasingly more complex chemistry. These systems are precursors of protocells that preceded the emergence of ancestral cells. (B) Putative representation of a protocell (adapted from [3]). Independently of the type of chemicals involved, e.g., pure RNA catalysts/”genetic” information or peptide/RNA, a protocell should contain three components: a compartment, a catalytic and energy harvesting machinery, and an information system. These components should work in an interconnected fashion to achieve the prolonged activity necessary for the protocell evolution. The interconnectedness in the systems is visible if one considers the various arrows between molecules/components: The catalytic machinery is defined and controlled by the information component (I) and the compartment (via encapsulation), whose molecular species are in turn produced by the catalytic machinery (II: information replication, III: amphiphile production, IV: energy harvesting and chemical replication, and V: catalyst amplification, which can lead to VI: replication process of the whole protocell). The compartment will also define the access of the protocell to environmental resources and, in part, the energy harvesting capabilities. It will also be instrumental in the replication (VI). It might also permit an interface-driven multiphase chemistry (see text below). Molecular precursors (i.e., resources to build protocell chemicals) are highlighted by black dotted structures or frames. Original chemicals of the protocell are highlighted by thick grey dotted frames. Products of the catalytic machinery are placed over a grey background. The involvement of catalysts is depicted by dashed arrows, that of information components with a plain arrow, and that of the compartment (expect the encapsulation) by dotted arrows. Note that the energy-related aspect would be involved in all chemical syntheses but, for the sake of clarity, is only shown once.

Research in the origins of life field or abiogenesis (emergence of life from non-life) attempts to answer a question that has fascinated humanity for millennia: Where do we come from? Whereas early attempts were more metaphysical in nature, insights into the nature of living systems with the discovery of cells as the basic unit of life and more recent advances in the understanding of the inner workings of its biochemistry have transformed the question into a scientific, empirical endeavor with two complementary goals. One is to explain of the emergence of contemporary cells through historical reconstruction, i.e., the construction of chemical models called protocells [1] (Figure 1); the other is to mimic cellular architectures to create artificial cell-like entities in relation with various applications that range from medicine to environmental remediation, over chemical/biological manufacturing [2].

**Figure 2:**
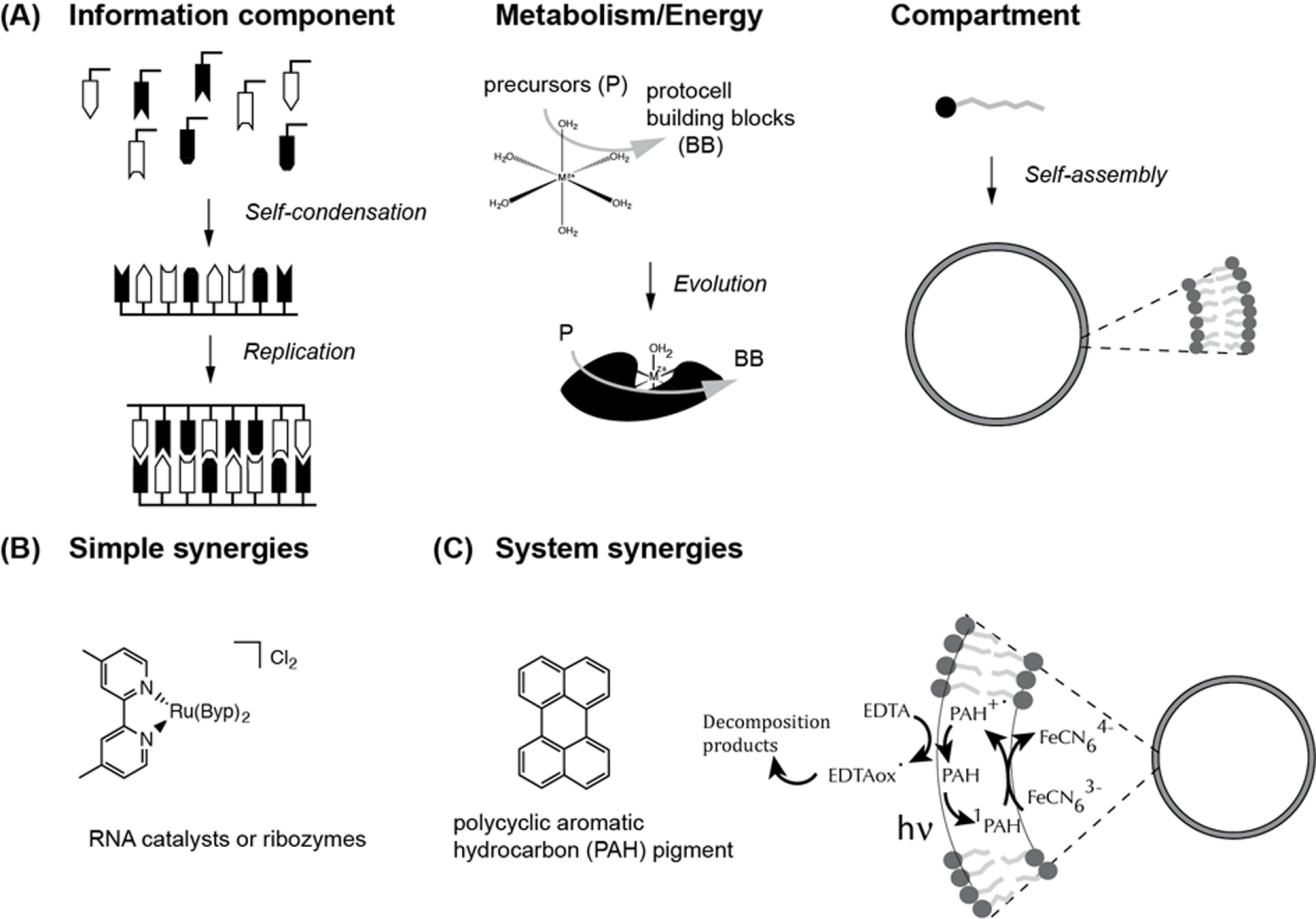
The bottom-up approach research strategies. (A) Each protocell component (vide infra) can be investigated in “isolation” to better understand the various processes pertaining to its synthesis/formation: information polymer, functional catalysts or self-assembly/stability of the compartment. (B) A higher degree of complexity can be attained by using chemicals that by themselves already link to two component functions: for instance, metal complexes that can harvest light (here a ruthenium tris(bipyridine) and catalyse reactions, or polymers such RNA ribozymes that are both genotype (information component) and phenotype (catalyst). (C) The systems approach offers insight into the increased level of cooperativity necessary to grasp the complexity of living interactions. The creation of chemical gradients, for example, requires the presence of a compartment and an energy harvesting system. In the case of PAHs, which are sparingly soluble in water, the compartment boundary not only allows for a distinction between two aqueous volumes, it also increases the availability of the PAH molecules by providing a specific hydrophobic environment for their solubilization, thereby improving the energy conversion.

The main challenge in the historical reconstruction is the scarcity of, occasionally even contradictory, information about i) the early Earth, both in terms of environmental conditions and chemical inventory, and ii) the putative transitions that must have been involved to convert a dynamic, molecularly diverse chemical environment into a coherent, interconnected network of chemical processes, leading ultimately to contemporary biochemistry. Even when a deconstructive (top-down) approach, i.e., the attempt to simplify the current biochemistry towards a simpler origin, is used, the fact that contemporary biomolecules and biochemical molecular assemblies, and their precursors themselves are likely optimized products of a long evolutionary process [4] renders this endeavor quite difficult. Hence, researchers in the field have tended to pursue alternative approaches in relation to the emergence of specific biomolecules and biochemical assemblies. The pursuance of such, normally parallel, approaches has led to the development of hypotheses either called by their chemical embodiment, such the lipid- [5], PAH- (polycyclic aromatic hydrocarbons) [6], and RNA-worlds [7], or designated by a general concept such as the metabolism- and gene-first scenarios [8]. This multi-faceted approach (Figure 2), whilst suffering somewhat from a lack of effective integration or cohesion, has nonetheless permitted the accumulation of essential insights in the characteristics of various biomolecules, e.g., the catalytic activity of RNAs and their evolution potential [9–11], as well as processes that were essential for their syntheses, such as Fischer–Tropsch-like reactions [12], non-enzymatic RNA [13] or peptide polymerization [14]. Moreover, it has also allowed for the determination of environmental conditions conducive to the self-assembly of several cellular-like components, such as bilayer membranes [15] and simple energy systems [16], or dynamic processes, such as growth and division [17–18] and potential evolution [19]. However, the experimental set-ups during these investigations have often been optimized to yield the best possible outcome rather than allow for chemical diversity and integration to “evolve” as a function of time, energy and molecular inputs.

This modular research mechanism, where themes are explored in relative isolation has clear limitations when these various “prebiotic” molecular systems are to be consolidated in a single protocell model. Moreover, situations emerge where one line of experimental enquiry becomes at odds with another feature that is equally integral to the whole. An example of this involves the selection of RNAs for catalytic activity, which often requires the presence of high ion concentrations that are disruptive for the formation of primitive membrane models. Membranes composed of putatively prebiotic amphiphiles, such as single hydrocarbon chain species [20–21] may have been exemplars of such membrane components. Furthermore, experimental conditions are sometimes implausible from the geochemical perspective. Finally, the evolutionary continuity of the systems, which should be paramount to explain the emergence of protocellular systems and evolution towards true cells, is often neglected in these experiments.

This short, necessarily selective, overview clearly underscores the necessity of new approaches, a fact that has led many researchers to propose the concept of chemical systems [22–23]. That is, the origin(s) of life, which is(are) hallmarked by the appearances of emergent properties (capacity of self-maintenance, self-replication and evolution under external constraints), should be investigated using a systemic approach where the functionalities in a chemical mixture are derived from the multiple interactions or “interconnected work” that exists between the various chemical processes. This approach has the advantage of allowing for the emergence of chemical interconnections between the various biomolecular classes, which should explain the deep interconnection between cellular subsystems, and implies the fact that the various molecular systems in cells might have co-evolved in relation to a specific geochemical environment. It also encompasses an important, often neglected, dimension: the fact that mixtures of disparate molecular classes imply a certain chemical contiguity in their syntheses. From the point of view of chemical research, a systems approach has, however, one obvious drawback: One should not expect the usual high reaction yields and chemical purity for the products. This fact highlights a fundamental difference in granularity of vision between traditional synthetic chemistry and systems chemistry in a prebiotic context. Whilst yield, purity, and conversion rates are key drivers of synthetic chemistry, those drivers for prebiotic systems chemistry appear to be less important than integration, contiguity, auto-catalysis and periodicity.

In this short article, we will first attempt at defining chemical systems and chemical contiguity. Then, using recent reports on chemical systems, we will highlight the potential of the “chemical system” approach for the investigation of the origin of pre-cellular systems and protocells.

### What are chemical systems?

Chemical systems are defined here as chemical mixtures comprising a network or set of interacting molecules. That is, system-dependent behavior and the system processes cannot be ascribed to any of the components acting in isolation. For instance, the catalysis by a metal complex in a bulk medium is inherently dependent on the nature of the chemicals (catalyst and substrates). However, if the catalysis is only possible in the presence of a third substance, not per-se involved in the catalytic process, but nevertheless necessary for it because it acts to organize the reactants, then one observes a chemical system. In a mathematical sense, chemical systems are sets or a collection of distinct objects/molecules, considered as an object in their own right.

Using this rather inclusive definition, a chemical system can be composed, in its simplest manifestation, of very few molecules also incorporating elements of their geochemical environment. At first glance, this definition seems too broad in terms of system composition. But the important aspect of the definition should in all cases remain the emergent properties, namely interconnectedness of the system and how the system behaves, rather than the contingent chemical composition of the system processes.

### What is chemical contiguity?

The notion of chemical systems also implies the existence of chemical contiguity. Many aspects of cellular biochemistry, e.g., in bioenergetics, glycolysis, the Krebs cycle or the intricate peptide formation systems, pre-suppose a form of chemical contiguity in their emergence. The Oxford English Dictionary defines contiguity as “the condition of touching or being in contact whether physical or non-physical”. In the chemical context employed here contiguity is seen as a connected gradient of physico-chemical conditions through which the different components of a chemical system (or “set” as above) can be synthesized and achieve their connectivity.

### System chemistry and chemical contiguity in the geochemical context

Geochemistry in conjunction with extra-terrestrial delivery of compounds must have defined not only the types of molecules that were present on the early earth, but also the molecular composition of early chemical systems and by extension that of protocells and contemporary cells. Furthermore, the environmental conditions must have defined the potential reactivity of these compounds. While these statements are agreed upon, the exact environmental parameters, i.e., chemical composition, temperature or availability of light energy, and the global geological make-up, for instance, a water-immersed mineral- [24] continent-island [25] or ice-covered earth [26] remain highly debated because of the lack of direct evidence. Interestingly, the experimental studies that attempt to link environmental conditions and chemical processes deemed essential for the emergence of life show that whatever the actual conditions, one can in many cases demonstrate that these diverse environments can foster comparable processes. In most cases, the type of chemistry envisioned can be categorized as heterogeneous catalysis [27] and ultimately periodic. There are reports of chemical synthetic continuity in aqueous solutions, but under conditions that seem to be unlikely in the geochemical context [28].

Thus, short of proposing a global, environmentally anchored solution to the syntheses of all molecules necessary for life to emerge [29], distinct geochemical environments could have not only produced specific chemicals, but could also have contributed to their evolution at different stages.

For instance, the idea of RNA polymers as information components, precursors of a genetic system, can be partially realized: Monomers can be efficiently polymerized in salt eutectic [30] and ice/water systems [31–32] or on mineral surfaces [33] or likely in porous mineral formations, i.e., formations that are presenting embedded channels or cavities within the minerals, where their accumulation has been suggested possible [34]. However, caution should be exercised over in extrapolating what is a computational study [34] to experimental scenarios. Moreover, the same environments are likely conducive to the function and evolution of these RNA polymers towards higher catalysis. In this case, direct evidence only exists for the eutectic phase in water/ice [35–37], but computer modelling [38] and preliminary wet-chemistry experiments, which show a selective accumulation of long oligomers [39], already hint at the possibility of similar processes taking place in mineral formations. In the same environments, short peptides, which are potential functional catalysts, can also be synthesized from simple amino acids [40]. Indeed, dipeptides can catalyse RNA oligomer formation in the eutectic phase of water/ice [41], underscoring another possible chemical contiguity within the geochemical context.

The ubiquity of polyphosphate in bioenergetics, but also of phosphate in cellular sensing and, in general, in the composition of some essential synthetic cellular products also suggest a common origin for the involvement of phosphate, that is, a form of synthetic contiguity [28]. This ubiquity of phosphoesters, mostly as phosphorus (P) in +5 oxidation state, is puzzling to some extent as this element is today a limiting nutrient for life [42]. But the prebiotic availability of P is now being far better understood [43–45]. In addition, the reactivity of phosphate and polyphosphate is low in aqueous media in the absence of catalysts, which affords a barrier to these species having been instrumental in the origins of life [7]. However, the reactivity of pyrophosphites (P with a +3 oxidation state) [46–47] is large enough to concomitantly permit phosphorylation reactions to activate small chemicals, as such as amino acids and permit their oligomerization, as well as to synthesize other compounds essential to life, such as amphiphiles, the proposed building blocks of prebiotic compartments, which can then self-assemble into vesicles under the same experimental conditions [48]. Pyrophosphites could thus be considered a common precursor energy currency for prebiotic catalysis, the activity of which is likely to be broader than these two chemical examples.

Mineral surfaces and porous matrices can also induce the formation of chemical systems of potential interest in the context of the origins of life. Several research groups have demonstrated their abilities to induce formation of evolved protocell systems. For instance, they have been shown to be capable of accumulating small molecules on their charged surfaces (electrostatic interactions) [49] or within pores and brines by thermophoresis and convection processes [50]. In the case of amphiphiles, these phenomena lead to the formation of compartments by self-assembly, which can encapsulate other solutes, e.g., RNA [17,51]. The accumulation ability of porous minerals allows for the amphiphile concentration to surpass their critical vesicle concentration to effect self-assembly [51]. Thus, mineral surfaces and porous formations could have been excellent media to foster the emergence of “self-contained”, dispersed chemical systems.

Furthermore, mineral surfaces can serve as supports for chemical systems to undergo organization. The polymerization of nucleic acid monomers has been achieved in this manner: When amphiphile vesicles or liposomes are dried in the presence of solutes on a silicate support, a system of stacked lipid bilayers with intercalated solutes is formed [52]. In this arrangement, the nucleotides are optimally spaced to react and form nucleic acid oligomers [53–55]. The presence of the mineral support is crucial here as it permits the preservation of the amphiphile bilayer structure during drying, thereby promoting the conversion of an “unreactive” organization (free floating vesicles and free monomers) into reactive chemical systems (stacks of alternating amphiphile bilayers and monomer layers). In stark contrast to the polymerization of RNA on montmorillonite, the absence of strong direct interactions between the mineral surfaces and the molecular species does neither reduce the chemical availability of the reaction products, nor preclude the “re”-dispersion of the lipid phases into dispersed aggregates with encapsulated catalysis products [52].

### Chemical systems and chemical contiguity in the dispersed state

The chemical systems aspect during the emergence of cell-like entities can also be highlighted once the chemical systems become dispersed; i.e., once a stage in chemical evolution is reached where self-propagating, chemically simple compartmentalized systems have emerged [56]. As mentioned earlier, the expectations when approaching the question of life origins from a chemical system point of view are related to the emergence of properties that are systemic in nature. The different properties can occur at various levels: i) Systems are able to segregate chemicals, thereby explaining why a class of molecules or specific molecules have been selected or discarded during chemical evolution; ii) systems are able to allow for the physical organization of molecules into functional catalytic/information networks; iii) systems foster evolutionary processes by maintaining chemicals in close proximity, that is, at physical distances permitting their further reactivity, while allowing for reaction wastes to be disposed of, and finally iv) systems could have conditioned the proliferation of functional systems.

#### Chemical selection

The investigation of synthetic pathways to biochemically relevant molecules has clearly underlined the need for some form of selection. Indeed, molecules of interest (nucleobases [57], sugars [58], amphiphiles [59]) are usually synthesized as minor products within a larger collection of derivatives even in the case of polymeric products, e.g., RNA analogues are formed with varying phosphodiester-bond regioselectivity [32]. The time frame in which this selection occurs is still uncertain, as are the “processes” that led to the selection. While the selection of fatty acids is undisputed as they are the main constituents of the hydrophobic core of modern membranes, their involvement in forming protocell compartments as the only type of amphiphiles can be disputed. Indeed, other amphiphiles or co-surfactants, if available via prebiotic syntheses [20,60–62], could have also contributed to the formation of primitive amphiphile-based structures, by allowing structure stabilization under prebiotic conditions, e.g., high ionic strength or temperature or stringent pH values.

Selective association of chemicals with fatty acid vesicles demonstrates that chemical systems, even simple ones, could have spawned such a selection by conditioning the interactions between their molecular constituents. For instance, canonical nucleobases interact more extensively with the vesicles structures than some of their derivatives and even stabilize them [63]. The same observation was made for ribose over other sugars. Moreover, when the permeability of fatty acid vesicle bilayers towards sugars was examined, ribose was determined to have the highest diffusion rates among aldopentoses or hexoses [64], a fact that could also explain its selection for the backbone of nucleic acids.

#### Catalysis support

The promotion of some complex catalyses was also shown to occur more readily in the presence of molecular assemblies, that is, in the context of a chemical system. Such effects could be either directly linked to the insertion into/association with the chemical system structure or to the encapsulation of a reaction “machinery” within it.

**Interface-linked catalysis:** The oligomerization of peptides from amino acids with condensing agents has been demonstrated to occur in the presence of phospholipid vesicles [65–67]. In these studies, the polymerization of hydrophobic amino acids was enhanced (in terms of yield and product length in monomer units), whereas that of hydrophilic, charged amino acids depended on the types of lipid headgroups used, i.e., whether ionic interactions could occur between amphiphile and amino acid. The authors surmised that the product length (up 29 monomer units compared to 9 in aqueous set-ups) was possible due to solubilization of the products within the hydrophobic core of the vesicle bilayers. Recent investigations with potentially prebiotic fatty acid structures have confirmed these observations [68]. In this case, the catalytic enhancement could be directly related to the protonation state of the acid function of the amphiphile head-groups.

Several studies also underscore the strength of the chemical systems approach in fostering complex catalysis and energy harvesting functions through association with the interface of chemical systems. For instance, the activity of an RNA polymerase ribozyme was improved when the various RNA compounds of the system (the ribozyme, the template/primer) were derivatized with amphiphilic moieties and co-associated within micelle structures [69]. Although no catalysis was demonstrated yet, amino acid and peptide-derivatized fatty acids (synthesized via a prebiotically plausible route) have been shown to associate with fatty acid vesicles. Vesicles with arginine-derivatized fatty acids could even electrostatically recruit RNA from the surrounding medium [70]. Such vesicles with associated ribozymes could eventually prove to be novel functional chemical systems.

The production of fatty acids from non-amphiphilic picolylesters performed using a photochemical reaction involving a ruthenium tris(bipyridine), functioning as photosensitizer and redox catalyst, and a nucleobase, 8-oxoguanine, serving as recyclable electron donor to trigger the redox cleavage of the precursor molecule, [71] was also found to be enhanced by the presence of pre-formed fatty acid vesicles. In aqueous media, both parts of the photochemical catalyst needed to be covalently linked (i.e., the intramolecular electron transfer was necessary for efficient conversion of the precursor), whereas when independently associated onto compartments they could work with the same efficiency via an intermolecular electron transfer [72]. Thus, the existence of chemical systems that incorporate boundaries with differing hydrophilicities and hydrophobicities could have enabled complex chemistries to emerge.

Energy harvesting from primary sources (light, geothermal, or chemical energy) and its conversion into chemical energy, such as proton and electron gradients or molecular energy currencies, is ubiquitous within contemporary biological cells. Thus, the emergence of such functions seems to be conditioned by the existence of chemical systems. Compartment models with their high molecular permeability [73] have long been considered an obstacle to the early emergence of energy harvesting apparatus. However, recent studies [16,61,74] have substantiated their potential early existence. Indeed, a class of photosensitive chemicals, the polycyclic aromatic hydrocarbons, PAHs (Figure 2), are capable of spontaneously inserting into amphiphile structures, even medium-length fatty acid vesicles (fatty acids with a hydrocarbon chain length of 8–12 carbon atoms), where they can drive the formation of proton [75] or electron gradients [16]. In the case of photo-induced electron transport over membranes, the differentiated permeability of small anionic solutes with high charge density, such as potassium ferricyanide, and EDTA used as an external sacrificial reductant was key to the reduction of the ferricyanide to ferrocyanide. Thus, simple compartments can harbor a directional charge transfer, induced by light harvesting.

By contrast, even though the formation of proton gradients upon the irradiation of bilayers into which PAHs have been incorporated has been reported [75], their dissipation is rapid. That is, utilization of the energy gradient should be directly linked to its formation. The build-up of the proton gradient underlines the importance of having a compartmentalization system. Indeed, the proton release upon irradiation of PAHs is not directional. Thus, only 50% of the protons generated will enter the lumen of the structures, the remainder being lost to the surroundings. However, the ensuing local concentration can result in transient pH gradients as large as three pH units, which could be large enough to couple a proton gradient to a reaction network (presumably as long as its dynamic stability is on a similar scale or longer to reaction rates).

Interestingly, while the presence of amphiphile structures acts to solubilize the highly hydrophobic PAHs, hence their light harvesting activity, the inserted PAH molecules in turn contributed to stabilizing the aggregates and reducing the bilayer permeability to additional small solutes [74]. That is, feedback interactions between system components significantly increase the probability of coupled functionality, in this case coupling of a light harvesting apparatus to chemical energy gradient formation.

**Volume-enclosed catalysis:** Compartmentalization of an aqueous volume within defined, preferably semi-permeable boundaries, was recognized very early on as paramount for the emergence of life [76]. Following the elucidation of the cellular membrane architecture, amphiphile vesicles or liposomes, became the main type of compartment models for the study of the origins of life, although other systems could also serve the very same purpose [77–81]. Besides the chemical continuity arguments, amphiphile bilayers offer a very fine-tuned permeability to solutes and allow for the insertion of chemical species in their hydrophobic cores, thereby enabling a multiphase chemistry.

This protocell development has focused on two types of processes required for self-maintenance and self-reproduction: the synthesis of protocell building blocks, such as amphiphiles and catalytic and information biopolymers, and the processes linked to protocell replication (see section “iii) Support of functional systems proliferation”) occasionally linked to uptake and conversion of energy from a primary source, such as light. From the evolutionary point of view, syntheses of catalytic and information biopolymers seemed to be central to the origin of life because of ubiquitous presence in every aspect of the cellular metabolism, hence their involvement in early stages of life emergence seemed to be necessary. In particular, the synthesis of RNA, because of the ability of RNA to catalyse reactions as well as encode the cellular information (each RNA in principle represents both a genotype and phenotype), was often singled out as the “only” approach to solve the famous “chicken–egg” dilemma [4,7]. However, as advocated here and elsewhere [4,7], the complexity of de novo RNA synthesis and its functional interconnection with other biopolymers in the cellular context question its early, single-handed role.

The polymerization of short RNA chains and peptides has been investigated within aqueous vesicle lumens as well as water/oil emulsions, and coacervates. Two types of catalysts, metal ions [21,82] and enzymes [77,83–85], have been utilized, the latter catalyst type to remedy the absence of true “prebiotic” catalysts, such short peptides and RNA enzymes. Nevertheless, all these experiments highlight crucial aspects for the development of protocellular „metabolism“.

Inspired by the non-enzymatic, template-directed RNA polymerization in bulk aqueous solutions [7] (the synthesis of a RNA using a primer/template system and magnesium ions as catalysts), the Szostak group [21,82] has demonstrated that RNA could be synthesized within mixed vesicles composed of several types of “prebiotic” fatty acids and co-surfactants. That is, the vesicles could have retained the primer/template system while activated monomers crossed the vesicle bilayers by passive diffusion. Similarly, amino acids could be dimerized within vesicles [86]. In related experiments, Chen et al. [87] established that an inorganic catalyst itself, magnesium ions, could be delivered to non-functional hammerhead ribozymes with consequent induction of activity (self-cleavage). The enzymatic reactions were conducted within vesicles formed by long chained fatty acids, such as octadecenoic acid (oleic acid) using polynucleotide phosphorylase (PNPase, whose activity under normal conditions leads to RNA degradation, but in the presence of ribonucleotide diphosphates, NDPs, can polymerize random RNA strands) [83] and Q-beta replicase [88]. In the PNPase experiments, the selective permeability of simple membranes was sufficient to permit an internalized synthetic or catalytic activity albeit at low yield and rate levels. However, both highlighted a different aspect of the compartmentalization: The use of aqueous metal ions could jeopardize the integrity of the compartment [20], and the compatibility of protein catalysts, presumably products of a long evolution, with the compartment building blocks could be problematic. Indeed, the use of decanoic acid vesicles completely inhibited the PNPase activity (unpublished observations), a clear support for a co-evolution of the various components of protocellular systems. The metal-sensitivity issue could be partially resolved using mixed amphiphile membranes [20] or trapping of the metal ions via complexation [21].

Uptake and transduction of energy (light, geothermal, or chemical energy) is essential to permit the emergence of truly (semi-)autonomous protocells [89] and as mentioned above requires a form of compartmentalization. The direct linking of the energy harvesting with chemical conversions, although likely one of the first forms of energy transduction, had limited applicability considering that the formation of a carbon–carbon bond is a two-electron process and that current biochemistry is hallmarked by energy storage and timely-defined consumption. It is therefore apposite to ponder on the question of the emergence of energy storage in the form of high-energy currency molecular systems. Some experimental evidence exists to support scenarios involving membranes as a central participant in energy harvesting and conversion into usable chemical energy, by creation of high-energy bonds in P compounds or other molecules. So far the energy harvesting in protocell models composed of fatty acid vesicles has, to the best of our knowledge, not been attempted yet. There is perhaps one notable exception [90], which, however, does not produce a phosphodiester bond. This might be due to the fact that the bioenergetics of P is intimately linked to the presence of sophisticated protein machinery for the harvesting of light itself, and its conversion to a proton gradient, as well as its dissipation by the formation of ATP. The question as to whether, and if so what, alternative molecular assemblies could have been developed as primitive energy currency systems remains open and a topic of considerable debate.

However, experiments have been carried out to reconstitute photosynthetic machinery in phospholipid liposomes [91–92] and polymersomes [93]. In these experiments, the use of photosensitizer triads or bacteriorhodopsin has allowed for the conversion of light energy into a proton gradient, which in turn could be utilized to power an ATP synthase to produce ATP from inorganic phosphate and ADP. In these systems, the “artificial” photosynthesis attained transduction levels that were comparable to those observed in cells, but in a completely artificial compartment. That such a complex dynamic system can be realized in artificial membranes is remarkable. The correct orientation of the various compounds was easily determined chemically, e.g., by derivatization of the triad photo-sensitizer with a charged group that defined which side of the molecule could insert into the hydrophobic core of the membranes [92]. However, a correct addition sequence during system preparation was still necessary and it speaks against a separate evolution of the system parts. In the case of fatty acid experiments [90], fatty acid vesicles were formed on/around titanium oxide particles and the irradiation of the photosensitizer powered the reduction of NAD^+^ to NADH using a mediator, rhenium bipyridine (a molecule similar to the ruthenium complex in Figure 2B).

A concomitant development (complexity increase) of membranes and light/energy harvesting/conversion systems can thus be seen as a prerequisite in the evolution of the ancestral bioenergetics en route to the sophisticated organisation of the contemporary one.

#### Support of functional systems proliferation

To achieve a “life”-like status, protocells should have been able not only to maintain themselves, but also to reproduce and change (evolve). The reproduction phase involves replication of all its internal content (metabolic networks and information component) within a chemical system while its compartment boundaries grow. This growth–reproduction phase is then subsequently followed by a division–reproduction event leading to the formation of two “daughter” systems.

The propensity of amphiphiles to integrate pre-existing structures [94–95] has been experimentally exploited either by adding more amphiphiles at a pace that prevents the de novo formation of novel structures [17] or by adding amphiphile precursors that had to be converted within the structures into amphiphilic molecules themselves [83,96–97]. However, two features that are potentially detrimental to the reproduction of functional protocells were recognized: a) Even in the presence of a metabolic model, the reproduction of the internal “metabolic” network and compartment boundaries must be linked to avoid the production of non-functional systems [98]; b) the spontaneous division of the growing systems was found difficult to achieve in a predictable way. Early experiments used extrusion methods (i.e., structures were physically pressed through filters with very small pores, a procedure that leads to structure re-sizing, thereby to the production of smaller, more numerous structures) as a way to model a division process mediated by external stresses [17]. Alternatively, the agitation of grown vesicles was sufficient to induce division [18].

To address the first issue, the idea of linking the growth and division of the compartment boundaries to the internal metabolic activity, was explored in various ways. Assuming that an efficient, internal reaction network would change the osmotic balance across the bilayers, Chen et al. [99] demonstrated that vesicles experiencing a stronger osmotic pressure across their bilayers were able to scavenge amphiphiles from other vesicles in an isotonic state. That is, they can grow at the expense of “non-functional” (isotonic) systems. This result whilst interesting seems to be difficult to envision in a natural setting as the difference in ionic strength needed to observe this result was quite large and the vesicle boundary permeability is known to be high. However, an internal chemical production can achieve similar results [86,100]. The formation of a hydrophobic dipeptide [86] for example led to growth of functional protocells at the expense of non-functional ones.

The division of vesicles could also be linked to an internal chemical reaction. In this case [101], the irradiation of membrane-located photosensitizers stimulated the formation of disulfide bonds in small hydrophilic molecules in the vesicle lumen, which then migrated subsequently into the boundaries provoking changes in the membrane packing and, ultimately, division.

### Relevance of chemical systems and chemical contiguity to the emergence of life

During the last fifty years, research on the emergence of life has focused mainly on exploring mechanisms for obtaining biochemicals and related functions under prebiotically plausible conditions. These chemicals were then considered indispensable for the emergence and evolution of cellular life, and were extensively studied using simple chemical reactions or selection schemes to evolve them and enable novel functions. Many insights were gained and have allowed for a better understanding of living systems or their components to emerge, even allowing for new aspects of biochemistry to be revealed, such as for example, the discovery of riboswitch activity in bacteria after their selection in the laboratory [102].

However, the knowledge gained has also highlighted some clear issues about this approach, in particular the question of compatibility between the various, required biochemicals, their plausibility within a prebiotic context and their capacity to remain active outside of the cellular environments [4]. Today, it seems clear that a change of paradigm is warranted, thus the idea of chemical systems and its corollary, chemical contiguity, which must be explored in relation to early earth geochemistry. Although this approach is not new per se (one can correctly argue that Oparin’s coarcervates were already chemical systems) [77], more recent “conscious” developments of this approach have already yielded some noteworthy successes, which augur rather well for the future of the field. Indeed, the integration of the various components of presumptive pre-cellular entities within single chemical models have led to the discovery of new dynamic couplings between chemicals within a chemical system that might explain how and why certain molecules or functions were selected during chemical evolution from a large inventory of molecules or possible chemical reactivities.

**Figure 3:**
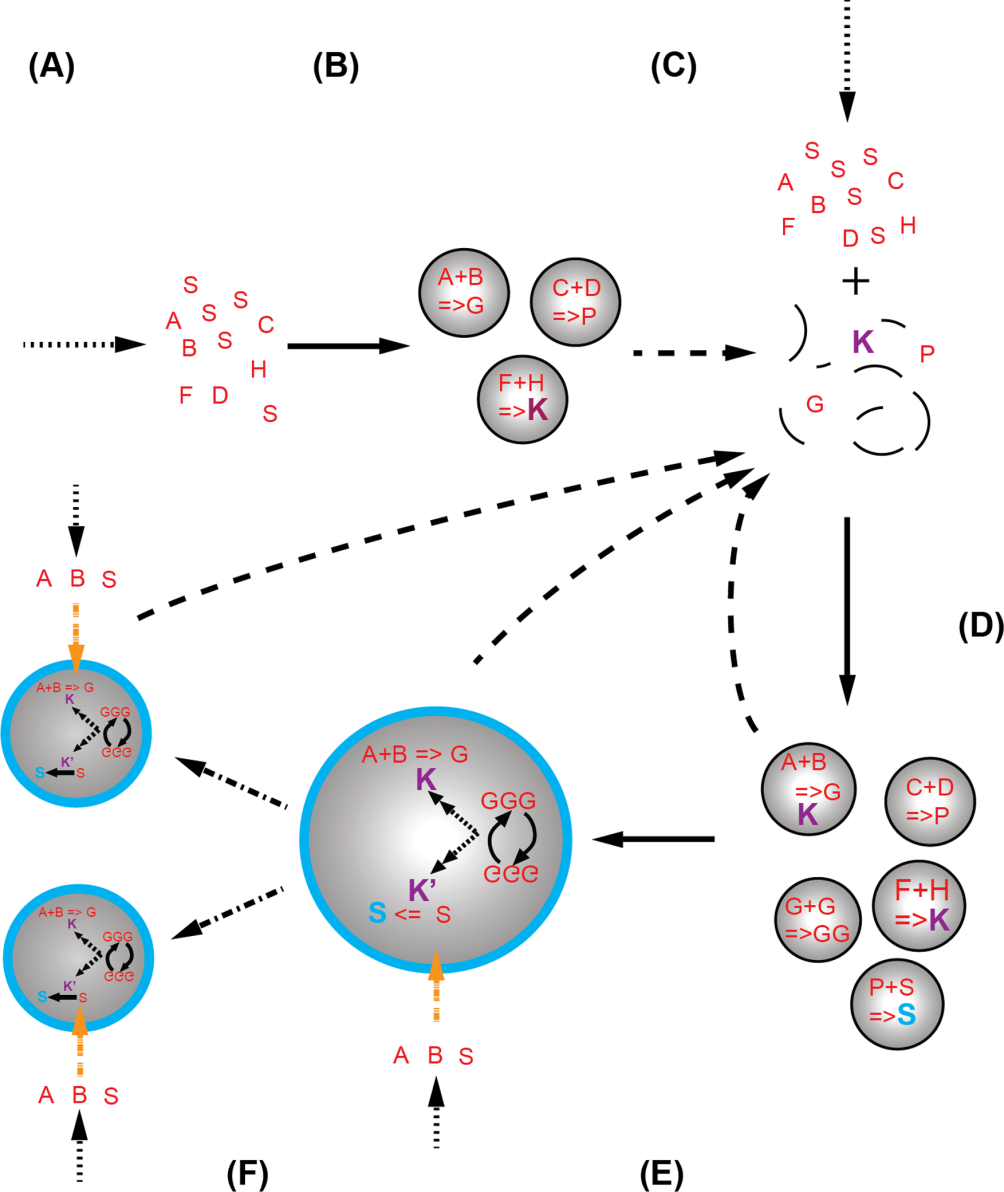
A putative scenario for the evolution of chemical systems towards protocells. (A) Prebiotic chemistry in the geochemical environment delivers an inventory of molecules (dotted arrow), some of which are amphiphiles (red S). When the aggregate critical concentration of the amphiphiles is reached, perhaps via trapping within a mineral pore structure, system compartments spontaneously form (plain arrow) and in the process co-locate chemicals, which could be either on the surface or within the system volume (B). The co-location allows a different chemistry (reactions are represented by letters with a “=>”) to take place as now both hydrophilic and hydrophobic environments contiguously co-exist. Upon subsequent disruption of the compartments (C) due to chemico-physical fluctuations (pH value, ionic strength, pressure or temperature) the prebiotic molecular inventory (dashed arrow) is enriched in a new set of basic building blocks, some of which “K” might be catalysts for the syntheses of building blocks of the system. Once the environmental conditions become again conducive to self-assembly, new chemical systems form (D). Some of them will have capability to produce further chemical complexity (new products or catalysed reactions). Cycles of formation/disruption will occur until (E) system compartments with improved stability (here highlighted by the blue boundaries composed of blue S, i.e., new amphiphiles) appear. These compartments will then gradually increase their internal catalytic network (dotted–dashed arrow) and gain some element of information processing capability, thus forming primitive protocells (Figure 1B). At that stage, they might still require chemical input from the environment (orange dotted arrow). However, they likely only take up certain chemicals selectively due to boundary permeability. These systems with increased half-life will perhaps also be disrupted cyclically until they are capable to self-replicate and adapt to environmental fluctuations (F). Once stable over long time periods, these systems would be clearly the first complete embodiment of a protocell (Figure 1B). Plain arrows relate to a self-assembly process, dotted arrows the prebiotic synthesis of chemicals, dashed arrows the disruption of a chemical system, the orange dashed arrows the selective permeability towards chemicals of the chemical system boundaries and the dotted–dashed arrows the replication process.

It is certain that some examples used as illustrations in this article are too artificial to have played any role in the actual evolution on the early Earth or are even altogether wrong. However, they underscore the potential of the chemical system approach to facilitate the study of the emergence of life and also document the work at hand. Its power lies in the variability of the concept that allows us to envision ever more complex systems, even consortia of them, which could have coalesced into protocells and later on ancestral cells (Figure 3). The main obstacle to that realization remains the fact that “dirty”, sub-optimal systems are difficult to understand with the rigor expected from chemistry.

## Conclusion

While it is obvious that the abiotic chemistry must have delivered the molecules needed for the emergence of cells or their precursors, the question about the transition between that abiotic chemistry and biochemistry remains unanswered. Many scenarios that often are referred to as “world” hypotheses have been proposed to explain that transition or its various stages, e.g., the lipid-, metabolism- or RNA-world, which in general tend to emphasize an aspect of the question that is directly related to the research field of their proponents. Each of these different, reductionist views is a natural one in the context of the Western scientific method. However, by electing to use a different granularity of vision, as by focusing on the system and what the system does, we can begin to explore connectivity of processes and how that integrates to system functionality. We expect these facets to be emergent in a molecular sense. Whilst they depend upon the specific chemical components used, it is how those chemicals integrate that leads to the function rather than any isolated property of the individual molecules themselves.

One of the chief historical features of the above origins hypotheses is their mutual exclusivity in respect of which chemical elements came first. However, a consensus is slowly building that co-emergence and co-evolution of the cellular functions must have started at an early stage. This hypothesis has resulted in a heightened focus on chemical systems in the field concerning the “Origin of Life”. Indeed, the study of complex molecular aggregates, which is now called “system chemistry” [103], seems to be consistent with the emergence of cellular complexity. Moreover, it has the potential to inherently satisfy the concept of evolutionary continuity. Obviously, an unambiguous demonstration is still necessary.
